# Analysis of Chemical Constituents of *Melastoma dodecandrum* Lour. by UPLC-ESI-Q-Exactive Focus-MS/MS

**DOI:** 10.3390/molecules22030476

**Published:** 2017-03-17

**Authors:** Jinfeng Wang, Ziyao Jia, Zhihao Zhang, Yutong Wang, Xi Liu, Linghua Wang, Ruichao Lin

**Affiliations:** 1 Beijing Key Laboratory for Quality Evaluation of Traditional Chinese Medicine, School of Chinese Materia Medica, Beijing University of Chinese Medicine, Beijing 100102, China; wangjinfengchn@163.com (J.W.); jzy646@163.com (Z.J.); tongjia_030si@163.com (Y.W.); spiritbucm@163.com (X.L.); 2 National Center for Natural Products Research, Department of BioMolecular Sciences, School of Pharmacy, University of Mississippi, Oxford, MS 38677, USA; sunnybucm@163.com; 3 Dongying Inspection Center for Food and Drug, Dongying 257091, Shandong, China; wlhdongying@163.com

**Keywords:** *Melastoma dodecandrum* Lour., ultra performance liquid chromatography, mass spectrometry, chemical constituents, identification

## Abstract

The ethnic drug *Melastoma dodecandrum* Lour. (MDL) is widely distributed throughout South China, and is the major component of Gong Yan Ping Tablets/Capsules and Zi Di Ning Xue San. Although the pharmacological effects of MDL have been well documented, its chemical profile has not been fully determined. In this study, we have developed a rapid and sensitive UPLC-ESI-Q-Exactive Focus-MS/MS method to characterize the chemical constituents of MDL in the positive and negative ionization modes. A comparison of the chromatographic and spectrometric data obtained using this method with data from databases, the literature and reference standards allowed us to identify or tentatively characterize 109 compounds, including 26 fatty acids, 26 organic acids, 33 flavonoids, six tannins, 10 triterpenoids, two steroids and six other compounds. Notably, 55 of the compounds characterized in this study have never been detected before in this plant. The information obtained in this study therefore enriches our understanding of the chemical composition of MDL and could be used in quality control, pharmacological research and the development of drugs based on MDL. In addition, this study represents the first reported comprehensive analysis of the chemical constituents of MDL.

## 1. Introduction

Traditional Chinese medicine (TCM) has been used for thousands of years to treat a variety of different diseases. Based on its theoretical therapeutic efficacy and wide range of clinical applications, TCM has received considerable interest from healthcare professionals, as well as those working towards the identification of new therapeutic agents for commercialization. In contrast to the pharmacological characteristics of single agent drugs, multicomponent drugs can exhibit synergistic pharmacological effects, through a “network” approach, where multiple compounds interact with multiple targets, pharmacokinetic or physicochemical synergisms in vivo with interdependent activities to achieve an improved optimal effect [1,2,3]. It is therefore essential to evaluate the chemical composition of each TCM, so that this information can be used to support further studies, such as drug effect, toxicity and metabolism studies.

*Melastoma dodecandrum* Lour. (MDL) is extensively distributed throughout the southern provinces of China, including Guizhou, Fujian, Zhejiang, Jiangxi and Yunnan. This plant is widely used for its medicinal properties by the Yao, Miao and She people, as well as several other minority groups. Modern pharmacological studies have shown that MDL exhibits several biological effects, including antihypoglycemic, hemostatic, analgesic, anti-inflammatory, blood lipid reducing, antioxidant and liver protection properties [4]. MDL has been used to treat a variety of different ailments, including dysmenorrhea, postpartum abdominal pain, metrorrhagia, leucorrhea, hematochezia, dysentery, carbuncle swollen and boils. To date, only 76 compounds have been isolated from MDL, including organic acids, flavonoids, triterpenoids and steroids [5,6,7,8,9,10,11,12,13]. However, much of the chemical composition of MDL remains unknown, making it difficult to rationalize its bioactivity or evaluate the safety of this material as a therapeutic agent. There is therefore an urgent need to develop an analytical method capable of determining the chemical composition of MDL. With this in mind, the aim of the current study was to establish a rapid and sensitive method for identifying the constituents of MDL. In this study, we used a Q-Exactive Focus MS/MS method to obtain high-resolution mass spectra of the different components. This method was proven to be an advanced, accurate and reliable tool for the comprehensive identification of compounds belonging to a wide range of structural classes [14,15,16,17,18,19,20,21]. Using this method, we tentatively identified a total of 109 compounds, highlighting the efficiency and accuracy of this new technique.

## 2. Results and Discussion

**Figure 1:**
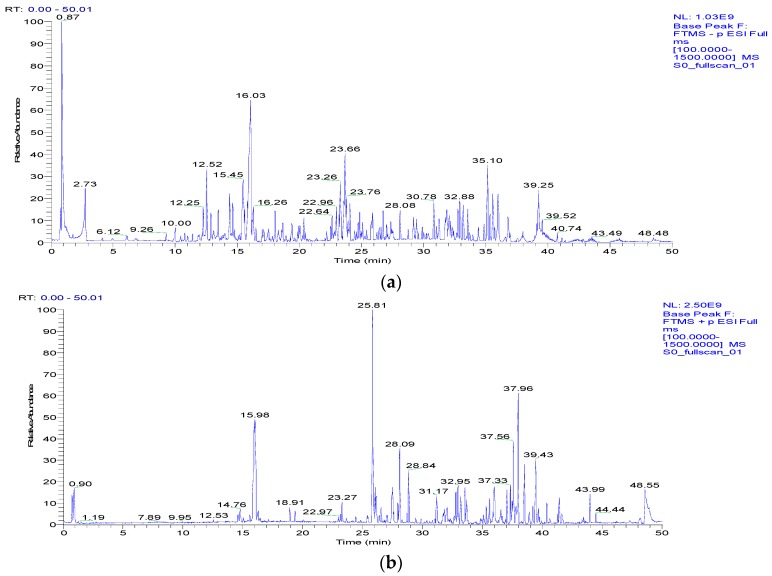
Base peak chromatogram of *Melastoma dodecandrum* Lour. in negative ion mode (**a**) and positive ion mode (**b**) using UPLC-ESI-Q-Exactive Focus-MS/MS.

*Melastoma dodecandrum* Lour. was analyzed in the positive and negative ionization modes using a Q Exactive Focus mass spectrometer, and the base peak chromatogram (BPC) chromatograms for both of these ESI modes are shown in Figure 1. Some of the compounds found in this study were identified based on a comparison of their analytical data (i.e., retention times and high-resolution mass spectra) with those of several reference standards. Thus compounds **10**, **34**, **35**, **46**, **68**, **79**, and **84** were unambiguously identified as gallic acid, luteolin, kaempferide, quercetin, oleanic acid, asiatic acid, and rutin, repectively. Moreover, the fragmentation patterns and pathways of the standards helped further confirm the structures of the derivatives of the reference compounds. Compounds without reference standards were identified by determining the elemental compositions of the precursor and product ions. The molecular formula and rational fragmentation patterns and pathways of these compounds were then identified based on a comparison of these data with chemical databases and the literature as described below in Section 3.5. In this way, we used a UPLC-ESI-Q-Exactive Focus-MS/MS method in combination with available standards, databases and literature data to characterize 109 compounds from MDL. Seven of these compounds were unambiguously identified based on a comparison with the corresponding reference standards. Data for all of these compounds are summarized in Table 1.

### 2.1. Fragmentation Pattern of Main Compounds

#### 2.1.1. Flavonoids

**Figure 2:**
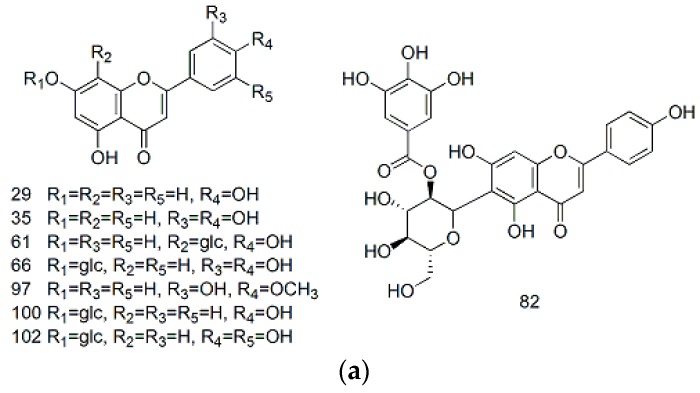

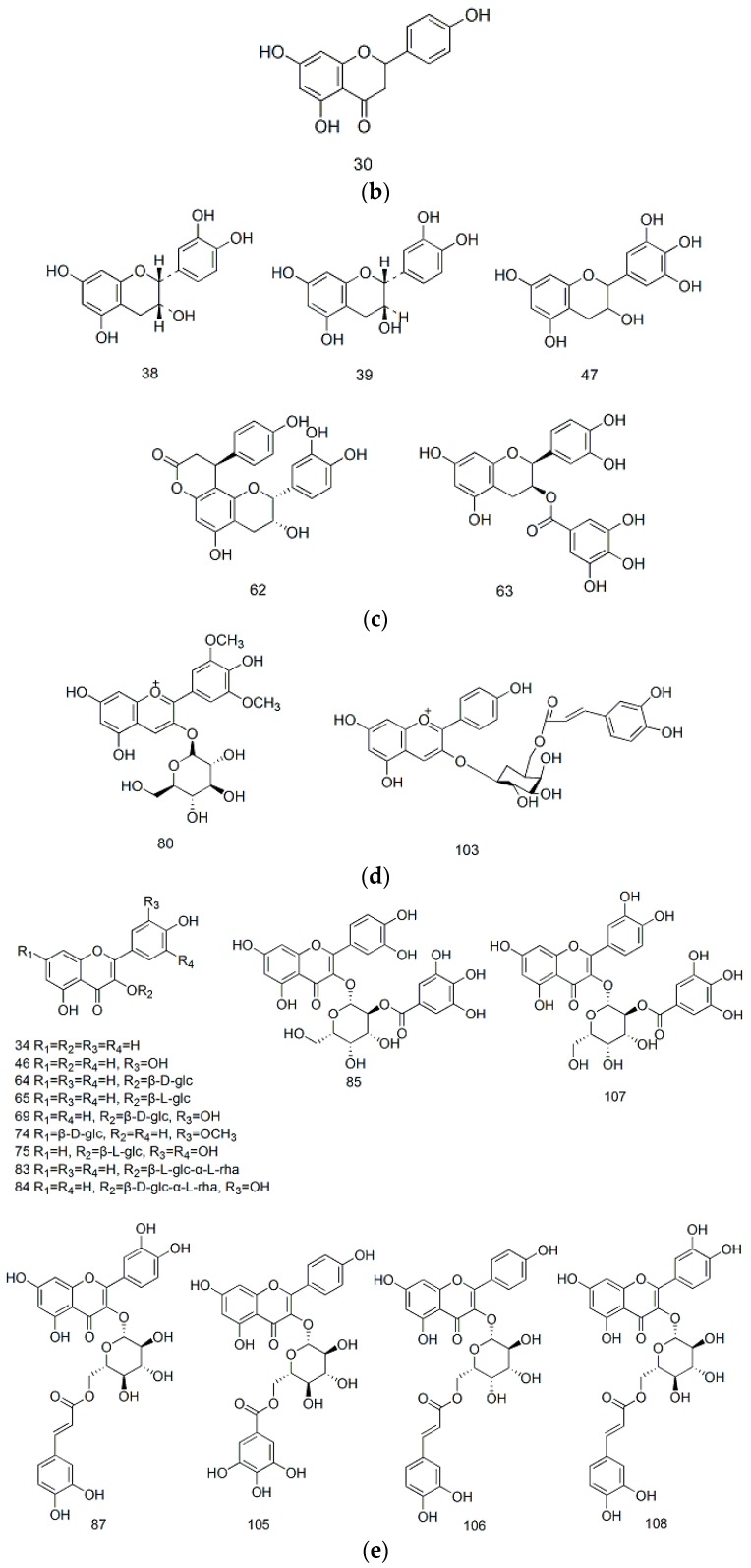
Structure of flavones (**a**); flavanones (**b**); flavan-3-ols (**c**); anthocyanidins (**d**); and flavonols (**e**).

Flavonoids are 2-phenylchromone systems that consist of two benzene rings (A and B) connected by a pyran ring (ring C), which is fused to the A ring. Flavonoids can be classified into several subclasses, including flavones, flavonols, flavanones, flavanonols, anthocyanidins, chalcones, isoflavonoids and flavan-3-ols, depending on the nature of the substituents attached to the different rings. Based on the results of accurate molecular mass measurements and the MS^2^ fragmentation pathways of the different materials [16,22,23], we characterized a total of 33 different flavonoids (aglycones) in MDL, including eight flavones, one flavanone, five flavan-3-ols, two anthocyanidins and 17 flavonols. The structures of the 33 flavonoids are shown below (Figure 2).

**Figure 3:**
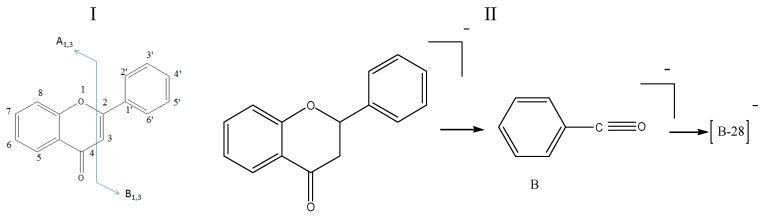
Fragmentation pattern I and II.

Two different fragmentation patterns and pathways were observed for the flavonoids (I and II, shown in Figure 3). The retro-Diels–Alder (RDA) fragmentation (I) would result in the formation of A 1, 3 and B 1, 3 as the main fragment ions of the flavonoid moiety (aglycones) because of the X 1, 3 cleavage of the C ring. The substituent groups on the parent compounds were determined based on the compositions of the A 1, 3 and B 1, 3 fragments. The fragmentation of the parent compound according to Pattern I resulted in high-intensity fragment ions, whereas the fragmentation according to pattern II result in low-intensity fragment ions. Furthermore, the main fragmentation patterns of flavonoids (glycosides) typically consist of fragments associated with deglycosylation, demethylation and decarboxylation yielding [M − H − Glc]^−^, [M − H − CH_3_]^−^ and [M − H − CO_2_]^−^ ions, respectively. The structures of the fragments resulting from the RDA fragmentation are shown in Table 2.

#### 2.1.2. Pentacyclic Triterpenes

**Figure 4:**
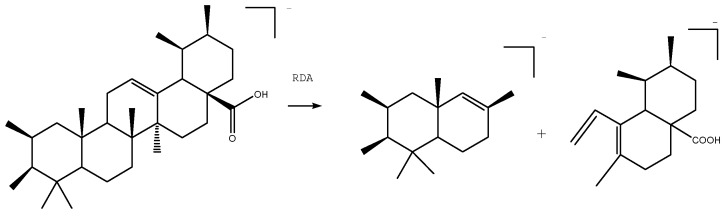
RDA fragmentation pathway of pentacyclic triterpene with endo-double bond.

**Figure 5:**
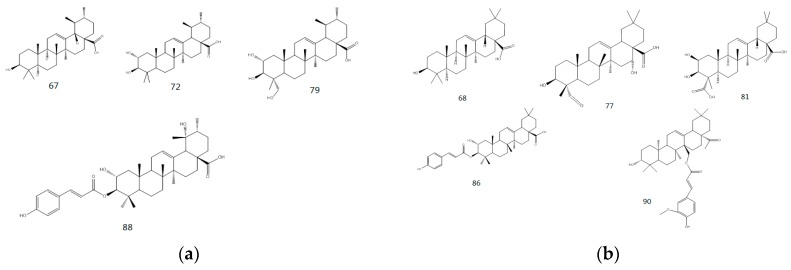
Structure of ursane-type (**67**, **72**, **79**, **88**) and oleanane-type (**68**, **77**, **81**, **86**, **90**) pentacyclic triterpene. This figure is fuzzy, please replace it with sharper figure.

Pentacyclic triterpenes are wildly distributed in Nature and consist of five rings, which are typically referred to as the A, B, C, D and E rings. Pentacyclic triterpenes can be divided into several different categories, depending on the nature of their E ring. In this study, we characterized two different types of pentacyclic triterpenes, including ursane- and oleanane-type pentacyclic triterpenes. The *endo*-double bond in pentacyclic triterpenes can readily undergo a RDA reaction during MS analysis (shown in Figure 4). Dehydration and decarboxylation are also observed as common fragmentation pathways in these systems during MS analysis [21,22,23]. Depending on the different substituents attached to their endo-double bonds and their accurate mass measurements, we were able to fully characterize all of the pentacyclic triterpenes found in MDL. As shown in Figure 5, we characterized a total of nine pentacyclic triterpenes using high-resolution MS^2^ mass spectrometry, including four ursane-type and five oleanane-type compounds pentacyclic triterpenes.

#### 2.1.3. Tannins

**Figure 6:**
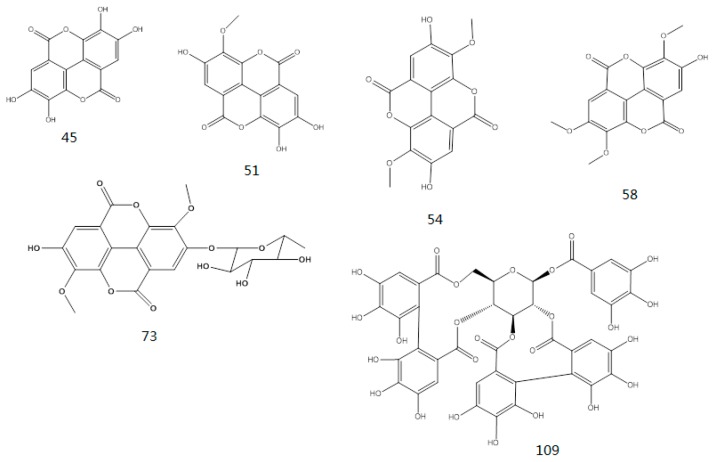
Structure of tannins.

The main structural features of tannins include their gallic acid ester moieties (or polymer) and glucose core (or other polyols). The MS^2^ spectra of the tannins revealed fragment ions with *m*/*z* values of 1091, 939, 769, 617, 599, 447 and 277. The *m*/*z* differences between these fragment ions were 152 and 170 atomic mass units (amu), which indicated that the fragments ions were produced by the successive removal of *O*-galloylhyperin and gallic acid anions. The fragment ion observed with an *m*/*z* value of 169 was characteristic of the fragment ions derived from *O*-galloylhyperin, whereas the fragment ion observed with an *m*/*z* value of 125 was attributed to the loss of CO_2_ from gallic acid [17]. A total of six other tannins were characterized in this way using high-resolution MS^2^ mass spectrometry. The structure of tannins characterized were shown in Figure 6.

**Figure 7:**
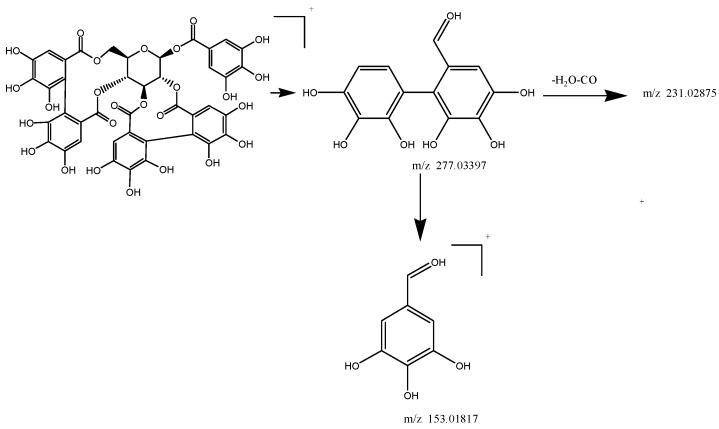
Fragmentation pathway of compound **109**.

Taking compound **109** as a representative example, the analysis of this compound in the positive ionization mode (*t_R_* = 12.61 min) gave an [M + H]^+^ ion with an *m*/*z* value of 937.09332, indicating a molecular formula of C_41_H_28_O_26_ (Δ = −0.89 ppm) (Table 2). The MS^2^ spectrum of compound **109** contained five fragment ions with *m*/*z* values of 345.02377, 277.03397, 231.02875, 171.04417 and 153.01817. The fragment pathway for this compound is shown in Figure 7. Based on these results, compound **109** was characterized as casuarinin.

#### 2.1.4. Organic Acids

Compound **4** (*t_R_* = 1.41 min) was analyzed in the negative ionization mode and gave an [M − H]^−^ ion with an *m*/*z* value of 147.02991, which indicated a molecular formula of C_5_H_8_O_5_ (Δ = 0.06 ppm) (Table 1). The MS^2^ spectrum of compound **4** gave three fragment ions with *m*/*z* values of 129.01924 [M − H − H_2_O]^−^, 101.02427 [M – H − HCOOH]^−^ and 85.02937 [M − H − H_2_O − CO_2_]^−^. This fragmentation process was used to confirm the identities of the other organic acids, resulting in the characterization of 26 compounds as organic acids.

## 3. Materials and Methods

### 3.1. Chemicals and Reagents

Acetonitrile and methanol (HPLC grade) were purchased from Fisher Scientific (Waltham, MA, USA). Distilled water was purchased from Watson’s Food & Beverage Co., Ltd. (Guangzhou, China). Formic acid (MS grade) was purchased from Fisher Scientific (Waltham, MA, USA). Reference standards of oleanolic acid (94.9%, batch No. 110709-201206), kaempferol (93.2%, batch No. 110861-201209), luteolin (100%, batch No. 111520-200504) and quercetin (97.4%, batch No. 100081-200907) were purchased from the National Institutes for Food and Drug Control (Beijing, China). A reference standard of asiatic acid (99%, batch No. 20150901) was purchased from CRM/RM center of China (Beijing, China). Reference standards of rutin (98%, batch No. 153-18-4) and gallic acid (98.5%, batch No. 149-91-7) were purchased from Chengdu-PUSH Bio-Technology Co., Ltd. (Chengdou, Sichuan, China).

### 3.2. Plant Materials and Sample Preparation

The whole plants of MDL were collected from Yunnan Province in China and authenticated by Professor Yaojun Yang (Beijing University of Chinese Medicine, Beijing, China). Voucher specimens (DN001) of the plant were deposited at the authors’ laboratory. The samples were dried and powdered, before being sieved through a 40-mesh sieve. A sample of the powder (approximately 2.0 g) was suspended in 25 mL of methanol, and the resulting mixture was subjected to ultrasonic treatment for 30 min before being cooled to room temperature. Methanol was added to compensate for the lost weight and the resulting mixture was filtered through a 0.22-μm PTFE syringe filter. The filtrate was collected and subjected to centrifugation (13,000 rpm, 10 min). The supernatant was then transferred to an autosampler vial for analysis by UPLC-ESI-Q-Exactive Focus-MS/MS.

### 3.3. UPLC-ESI-Q-Exactive Focus-MS/MS Analysis

UPLC analysis was performed on a Thermo Scientific Ultimate 3000 system (Sunnyvale, CA, USA) equipped with a binary solvent delivery manager and a sample manger. Chromatographic separations were performed on a Thermo Scientific Hypersil GOLD C18 column (100 mm × 2.1 mm, 1.9 μm). The column temperature was maintained at 40 °C. A mobile phase consisting of 0.1% formic acid in water (A) and acetonitrile (B) was used to elute the column according to the optimized gradient program, as follows: 98% A from 0 to 5 min; 98%–80% A from 5 to 15 min; 80%–40% A from 15 to 30 min; 40%–2% A from 30 to 40 min; 2% A from 40 to 47 min; 2%–98% A from 47 to 47.1 min; 98% A from 47.1 to 50 min. The flow rate was set at 0.3 mL/min. An injection volume of 5 μL was used for the reference compounds and then analytical samples. For MS detection, the operating parameters were as follows: spray voltage, +3500 V/–3200 V; atomization temp, 350 °C; sheath gas pressure, 35 arb; aux gas pressure, 10 arb; capillary temperature, 320 °C; S-lens RF, 60 V; resolution, MS full scan 70,000 FWHM, MS/MS full scan 15,000 FWHM; scan range, *m*/*z* 100–1500 for MS; *m*/*z* 30–1500 for MS/MS; scanning mode, fullscan-ddms2.

### 3.4. Optimization of Analytical Conditions

To obtain better chromatographic separation and mass spectrometric detection, we evaluated three different mobile phase systems, including aqueous methanol, aqueous acetonitrile and aqueous acetonitrile-formic acid solutions. The aqueous acetonitrile solution resulted in the best separation of the major components of MDL. Furthermore, the addition of 0.1% formic acid to this mobile phase resulted in a considerable improvement in the symmetry properties of most of the chromatographic peaks. We also varied the flow rate (0.25, 0.3, and 0.35 mL/min), column temperature (30, 35, and 40 °C) and injection volume (2, 3, and 5 μL) during method development. The results of these optimization experiments established the following conditions for the chromatographic separation of the different components of MDL: mobile phase, aqueous acetonitrile containing 0.1% formic; flow rate, 0.3 mL/min; column temperature, 40 °C; and injection volume, 5 μL.

### 3.5. Structure Analysis Procedure

In the positive and negative scan mode, based on the high-accuracy precursor ions and product ions obtained from Q-Exactive Focus-MS/MS, the elemental compositions were calculated when the maximum tolerance of mass error for all the precursor ions and product ions was set at 1.5 ppm, which can satisfy the requirements for positive identification. Based on the elemental compositions of the precursors, the most rational molecular formula was sought in different chemical databases such as the Spectral Database for Organic Compounds SDBS (http://sdbs.db.aist.go.jp), *m*/*z* cloud (https://www.mzcloud.org) and ChemSpider (http://www.chemspider.com). Meanwhile by searching literature sources, such as PubMed of the U.S. National Library Medicine and the National Institutes of Health, Scifinder Scholar of the American Chemical Society, Science Direct of Elsevier and Chinese National Knowledge Infrastructure (CNKI) of Tsinghua University, all components reported in the literatures on MDL and plants of the same family were summarized in a Microsoft Office Excel table to establish an in-house library [5,6,7,8,9,10,11,12,13] for searching the most rational molecular formula. When several matching compounds with the same formula were found, the fragmentation patterns and pathways of the compounds were analyzed and then validated by Mass Frontier 7.0 (Thermo Scientific) for positive identification.

## 4. Conclusions

A new UPLC-ESI-Q-Exactive Focus-MS/MS method was developed to analyze the chemical constituents of MDL based on their mass spectral fragmentation patterns. This new method resulted in the characterization of 109 compounds. The results of this study therefore provide an important reference to improve our understanding of the composition of MDL. We found that flavonoids are the main components of MDL, especially the flavonols, which possess a wide range of interesting pharmacological activities, such as anticancer, antibacterial, and antiviral activities. In terms of their structural characteristics, the triterpenoids found in MDL were ursane- and oleanane-type systems. Several tannins and steroids were also found in MDL. In addition to the fatty acids found in MDL, we found 55 other compounds that have never been reported in MDL besides fatty acids. Further studies pertaining to the chemical constituents in *Melastoma dodecandrum* Lour. are currently underway in our laboratory. Moreover, The study shows that, with the application of the UPLC-ESI-Q-Exactive Focus-MS/MS to characterizing the constituents of MDL, this method offers a rapid, sensitive and high throughput methodology for the identification of constituents of TCM prescriptions and herbal medicines.

## Figures and Tables

**Table 1:** Tentative identification of the chemical constituents of *Melastoma dodecandrum* Lour. by UPLC-ESI-Q-Exactive Focus-MS/MS in negative and positive modes.

No.	Tentative Compound	*t*_R_ (min)	Molecular Formula	Measured *m*/*z*	*m*/*z* Error in ppm	MS/MS (*m*/*z*)	Type of Compounds
**1**	Malic acid	0.99	C_4_H_6_O_5_	133.01422 [M−H]^−^	−0.19	115.00355 [M − H − H_2_O]^−^ 71.0138 [C_3_H_3_O_2_]^−^ 89.02435 [M − H − CO_2_]^−^	A
**2**	Salicylic acid	9.34	C_7_H_6_O_3_	137.02438 [M − H]^−^	−0.25	93.03443 [M – H − CO_2_]^−^	B
**3**	*m*-Salicylic acid	10.00	C_7_H_6_O_3_	137.02437 [M − H]^−^	−0.36	93.03445 [M – H − CO_2_]^−^	B
**4**	Citramalic acid	1.41	C_5_H_8_O_5_	147.02991 [M − H]^−^	0.06	129.01924 [M – H − H_2_O]^−^ 101.02427 [M – H − HCOOH]^−^ 85.02937 [C_4_H_5_O_2_]^−^	A
**5**	Protocatechuic acid	5.55	C_7_H_6_O_4_	153.01932 [M − H]^−^	−0.09	109.02937 [M – H − CO_2_]^−^	B
**6**	Gentisic acid	9.51	C_7_H_6_O_4_	153.01932 [M − H]^−^	−0.09	109.02943 [M − H − CO_2_]^−^ 108.02159 [C_6_H_4_O_2_]^−^	B
**7**	Pimelic acid	13.87	C_7_H_12_O_4_	159.0663 [M − H]^−^	0.11	115.07629 [M – H − CO_2_]^−^ 97.06577 [C_6_H_9_O]^−^ 141.0556 [M − H − H_2_O]^−^	A
**8**	Coumaric acid	13.94	C_9_H_8_O_3_	163.04005 [M − H]^−^	−0.08	119.05009 [M − H − CO_2_]^−^	B
**9 ^#^**	Vanillic acid	11.72	C_8_H_8_O_4_	167.03494 [M − H]^−^	−0.23	152.0114 [M − H − CH_3_]^−^ 123.045 [M − H − CO_2_]^−^ 108.02161 [C_6_H_4_O_2_]^−^	B
**10** *^,^**^#^**	Gallic acid	2.23	C_7_H_6_O_5_	169.01416 [M − H]^−^	−0.51	125.02429 [M − H − CO_2_]^−^ 97.0294 [C_5_H_5_O_2_]^−^ 81.03452 69.03456 [C_4_H_5_O]^−^	B
**11**	Shikimic acid	1.15	C_7_H_10_O_5_	173.04547 [M − H]^−^	−0.44	155.03479 [M − H − H_2_O]^−^ 137.02423 [M – H − 2H_2_O]^−^ 111.04502 [M − H − H2O − CO_2_]^−^ 93.03445 [C_6_H_5_O]^−^ 73.02941 [C_3_H_5_O_2_]^−^	B
**12**	2-Isopropylmalic acid	11	C_7_H_12_O_5_	175.06120 [M − H]^−^	0.03	157.05048 [M − H − H_2_O]^−^ 115.03992 [C_5_H_7_O_3_]^−^ 113.06075 [C_6_H_9_O_2_]^−^ 85.0658 [C_5_H_9_O]^−^	B
**13**	Glucose	0.89	C_6_H_12_O_6_	179.05605 [M − H]^−^	−0.37	59.01379 [C_2_H_3_O_2_]^−^ 71.01382 [C_3_H_3_O_2_]^−^ 89.02422 [C_3_H_5_O_3_]^−^ 101.02422 [C_4_H_5_O_3_]^−^	F
**14**	2-Hydroxy-3-(2-hydroxyphenyl)propanoic acid	9.78	C_9_H_10_O_4_	181.05064 [M − H]^−^	0.06	163.03993 [M − H − H_2_O]^−^ 135.04512 [M – H − HCOOH]^−^ 119.0501 [M − H − H_2_O − CO_2_]^−^	B
**15 ^#^**	Methyl gallate	10.88	C_8_H_8_O_5_	183.02988 [M − H]^−^	−0.11	168.00624 [M − H − CH_3_]^−^ 140.0114 [M − H − CH_3_ − CO]^−^ 124.01643 [C_6_H_4_O_3_]^−^	B
**16**	Citric acid	0.99	C_6_H_8_O_7_	191.01971 [M − H]^−^	−0.06	111.00864 [M − H − H_2_O – COOH − OH]^−^ 87.00864 [C_3_H_3_O_3_]^−^ 129.01915 [M − H − H_2_O − CO_2_]^−^ 85.02939	A
**17 ^#^**	Ferulic acid	18.04	C_10_H_10_O_4_	193.05052 [M − H]^−^	−0.57	178.02702 [M − H − CH_3_]^−^ 149.06059 [M − H − CO_2_]^−^ 134.03719 [M − H − CH_3_ − CO_2_]^−^	B
**18 ^#^**	Vanillylmandelic acid	14.47	C_9_H_10_O_5_	197.04573 [M − H]^−^	0.93	153.05563 [M − H − CH_3_]^−^ 138.03203 [M − H − CH_3_-CO_2_]^−^ 121.0294 [M − H − CH_3_ − CO_2_ − OH]^−^	B
**19**	Sebacic acid	20.14	C_10_H_18_O_4_	201.11327 [M − H]^−^	0.17	183.10249 [M − H − H_2_O]^−^ 139.11273 [M − H − H_2_O − CO_2_]^−^	A
**20**	1-Oxo-1,2,4-butanetricarboxylic acid	1.48	C_7_H_8_O_7_	203.01970 [M − H]^−^	−0.13	141.01923 [M − H − H_2_O − CO_2_]^−^ 97.02934 [M − H − H_2_O − 2CO_2_]^−^ 69.03453 [C_4_H_5_O]^−^	A
**21**	Undecanedioic acid	22.12	C_11_H_20_O_4_	215.12874 [M − H]^−^	−0.67	197.11826 [M − H − H_2_O]^−^ 153.12842 [M − H − H_2_O − CO_2_]^−^	A
**22**	2-Hydroxysebacic acid	16.5	C_10_H_18_O_5_	217.10825 [M − H]^−^	0.46	199.09734 [M − H − H_2_O]^−^ 171.10257 [M – H − HCOOH]^−^ 155.10768 [M − H − H_2_O − CO_2_]^−^	A
**23**	Glucoheptonic acid	0.82	C_7_H_14_O_8_	225.06168 [M − H]^−^	0.38	179.05602 [C_6_H_1_1O_6_]^−^ 161.04546 [C_6_H_9_O_5_]^−^ 87.00864 [C_3_H_3_O_3_]^−^	A
**24**	Traumatic Acid	22.97	C_12_H_20_O_4_	227.12894 [M − H]^−^	0.24	183.13876 [M − H − CO_2_]^−^ 165.12823 [M − H − H_2_O − CO_2_]^−^	A
**25**	1-*O*-galloyl-glycerol	8.17	C_10_H_12_O_7_	243.05095 [M − H]^−^	−0.32	169.01408 [M − H − CO_2_ − 2CH_3_]^−^ 125.02431 [M – H − 2CO_2_ − 2CH_3_]^−^	B
**26**	Oxododecanedioic acid	18.35	C_12_H_20_O_5_	243.12381 [M − H]^−^	0.05	225.1131 [M − H − H_2_O]^−^ 207.10254 [M − H − H_2_O]^−^ 181.1234 [C_11_H_17_O_2_]^−^	A
**27**	Palmitic acid	39.09	C_16_H_32_O_2_	255.23297 [M − H]^−^	0.07	237.06160 [M − H − H_2_O]^−^	A
**28**	Abscisic acid	19.75	C_15_H_20_O_4_	263.12885 [M − H]^−^	−0.12	219.13887 [M − H − CO_2_]^−^ 204.11528 [M − H − CO_2_ − CH_3_]^−^ 151.07634 [C_9_H_11_O_2_]^−^	B
**29 ^#^**	Apigenin	19.79	C_15_H_10_O_5_	269.04559 [M − H]^−^	0.17	117.03447 [C_8_H_5_O]^−^ 151.00354 [C_7_H_3_O_4_]^−^ 107.01373 [C_6_H_3_O_2_]^−^	C
**30 ^#^**	Naringenin	21.35	C_15_H_12_O_5_	271.06122 [M − H]^−^	0.08	177.01917 [C_9_H_5_O_4_]^−^ 151.00352 [C_7_H_3_O_4_]^−^ 119.05009 [C_8_H_7_O]^−^	C
**31**	Hydroxyhexadecanoic acid	36.77	C_16_H_32_O_3_	271.22797 [M − H]^−^	0.36	225.22221 [M – H − HCOOH]^−^	A
**32**	Oleic acid	39.49	C_18_H_34_O_2_	281.24860 [M − H]^−^	−0.01	237.06163 [M − H − CO_2_]^−^	A
**33**	Stearic acid	41.08	C_18_H_36_O_2_	283.26425 [M − H]^−^	−0.01	265.14810 [M − H − H_2_O]^−^ 237.06181 [M – H − HCOOH]^−^	A
**34** *^,**#**^	Kaempferol	19.95	C_15_H_10_O_6_	285.04047 [M − H]^−^	0.02	257.04535 [M − H − CO]^−^ 241.05112 [M − H − CO_2_]^−^ 151.00352 [C_7_H_3_O_4_]^−^ 133.02942 [C_8_H_5_O_2_]^−^	C
**35** *^,**#**^	Luteolin	21.79	C_15_H_10_O_6_	285.04062 [M − H]^−^	0.56	239.03464 [M − H − CO − H_2_O]^−^ 185.06078 [C_12_H_9_O_2_]^−^ 159.04491 [C_10_H_7_O_2_]^−^ 93.03454 [C_6_H_5_O]^−^	C
**36**	Hexadecanedioic acid	30.38	C_16_H_30_O_4_	285.20709 [M − H]^−^	−0.14	267.19635 [M − H − H_2_O]^−^ 223.20653 [M − H − H_2_O − CO_2_]^−^	A
**37**	3,5-Dihydroxy-hexadecanoic acid	23.84	C_16_H_32_O_4_	287.22278 [M − H]^−^	−0.01	269.21246 [M − H − H_2_O]^−^ 241.21735 [M − H − H_2_O − CO_2_]^−^	A
**38**	Epicatechin	11.49	C_15_H_14_O_6_	289.07193 [M − H]^−^	0.58	245.0816 [M − H − CO_2_]^−^ 203.07123 [C_12_H_11_O_3_]^−^ 137.02423 [C_7_H_5_O_3_]^−^ 109.02934 [C_6_H_5_O_2_]^−^	C
**39**	Catechin	13.27	C_15_H_14_O_6_	289.07184 [M − H]^−^	0.27	245.0089 [M – H − C_3_H_8_]^−^ 217.01398 [M − H − C_3_H_8_ − CO]^−^ 189.01923 [M − H − C_3_H_8_ − 2CO]^−^ 173.0242 [C_10_H_5_O_3_]^−^ 145.0294 [M – H − C_3_H_8_ − 2CO − CO_2_]^−^	C
**40**	4,9-Dihydroxy-6,7-dimethoxynaphtho(2,3-*d*)-1,3-dioxole-5,8-dione	14.56	C_13_H_10_O_8_	293.03040 [M − H]^−^	0.36	249.04028 [M − H − CO_2_]^−^ 225.11308 [C_12_H_17_O_4_]^−^ 162.0321 [M − H − CO_2_ − CH_3_ − CO]^−^	F
**41**	9-Hode	32.28	C_18_H_32_O_3_	295.22791 [M − H]^−^	0.12	277.21716 [M − H − H_2_O]^−^ 171.1026 [C_9_H_15_O_3_]^−^	A
**42**	Ricinoleic acid	32.63	C_18_H_34_O_3_	297.24353 [M − H]^−^	0.0	183.13885 [C_11_H_19_O_2_]^−^	A
**43**	2-Glucopyranosyloxybenzoic acid	9.95	C_13_H_16_O_8_	299.07730 [M − H]^−^	0.2	137.02425 [M − H − Glc]^−^ 93.03445 [M − H − glc − CO_2_]^−^	B
**44**	Hydroxystearic acid	39.09	C_18_H_36_O_3_	299.25919 [M − H]^−^	0.06	253.25348 [M − H − HCOOH]^−^ 225.22246 [C_15_H_29_O]^−^	A
**45 ^#^**	Ellagic acid	15.56	C_14_H_6_O_8_	300.99899 [M − H]^−^	0	283.99619 [M − H − OH]^−^ 245.009 [C_12_H_5_O_6_]^−^ 229.01402 [M − H − CO_2_ − CO]^−^ 201.01927 [M − H − CO_2_ − 2CO]^−^ 185.02431 [C_11_H_5_O_3_]^−^	D
**46** *^,**#**^	Quercetin	19.95	C_15_H_10_O_7_	301.03534 [M − H]^−^	−0.12	178.99843 [C_8_H_3_O_5_]^−^ 151.00351 [M – H − C_6_H_6_ − CO_2_ − CO]^−^ 107.01373 [M – H − C_6_H_6_ − 2CO_2_ − CO]^−^	C
**47**	Gallocatechin	11.12	C_15_H_14_O_7_	305.06683 [M − H]^−^	0.52	261.0766 [M − H − CO_2_]^−^ 219.06612 [C_12_H_11_O_4_]^−^ 167.03488 [C_8_H_7_O_4_]^−^ 137.02432 [C_7_H_5_O_3_]^−^ 125.02433 [C_6_H_5_O_3_]^−^	C
**48**	Eicosanoic acid	42.84	C_20_H_40_O_2_	311.29562 [M − H]^−^	0.22	293.17941 [M − H_2_O]^−^	A
**49**	Glucovanillin	10.72	C_14_H_18_O_8_	313.09308 [M − H]^−^	0.6	161.04539 [C_6_H_9_O_5_]^−^ 113.02431 [C_5_H_5_O_3_]^−^ 101.02427 [C_4_H_5_O_3_]^−^ 71.01381 [C_3_H_3_O_2_]^−^	F
**50**	Octadecanedioic acid	29.15	C_18_H_34_O_4_	313.23849 [M − H]^−^	0.2	295.2272 [M − H − H_2_O]^−^	A
**51 ^#^**	2,3,8-Trihydroxy-7-methoxychromeno[5,4,3-cde]chromene-5,10-dione	21.54	C_15_H_8_O_8_	315.01489 [M − H]^−^	0.80	300.99841 [C_14_H_5_O_8_]^−^ 269.10269 161.04578 [C_6_H_9_O_5_]^−^ 71.01388 [C_3_H_3_O_2_]^−^	D
**52**	Dihydroxystearic acid	30.79	C_18_H_36_O_4_	315.25403 [M − H]^−^	−0.17	297.24344 [M − H − H_2_O]^−^ 201.11363 [M − H − C_8_H_18_]^−^	A
**53**	Digallate	11.39	C_14_H_10_O_9_	321.02530 [M − H]^−^	0.29	169.01404 [C_7_H_5_O_5_]^−^ 125.02428 [C_6_H_5_O_3_]^−^	B
**54**	2,3-Di-*O*-methylellagic acid	20.25	C_16_H_10_O_8_	329.03040 [M − H]^−^	0.32	314.00681 [M − H − CH_3_]^−^ 298.98325 [C_14_H_3_O_8_]^−^ 270.98834 [M − H − 2CH_3_ − CO]^−^	D
**55**	Woodorien	9.46	C_14_H_18_O_9_	329.08795 [M − H]^−^	0.44	167.03482 [M − H − Glc]^−^ 152.01135 [M − H − Glc − CH_3_]^−^ 121.0294 [M − H – Glc − HCOOH]^−^ 108.02159 [M − H − Glc − CH_3_ − CO_2_]^−^	B
**56**	Galloylglucose	3.25	C_13_H_16_O_10_	331.06729 [M − H]^−^	0.67	271.04575 [C_11_H_11_O_8_]^−^ 211.02464 [C_9_H_7_O_6_]^−^ 169.01405 [M − H − Glc]^−^ 125.02432 [C_6_H_5_O_3_]^−^	B
**57**	Caffeic acid-3-glucoside	11.08	C_15_H_18_O_9_	341.08798 [M − H]^−^	0.52	305.06638 [M – H − 2H_2_O]^−^ 281.06647 [C_13_H_13_O_7_]^−^ 251.05588 [C_12_H_11_O_6_]^−^ 221.04532 [C_11_H_9_O_5_]^−^ 179.03485 [M − H − Glc]^−^ 135.04509 [M − H − Glc − CO_2_]^−^	B
**58**	2-Hydroxy-3,7,8-trimethoxychromeno[5,4,3-cde]chromene-5,10-dione	23.33	C_17_H_12_O_8_	343.04590 [M − H]^−^	−0.13	328.0224 [M − H − CH3]^−^ 312.99899 [M – H − 2CH_3_]^−^ 297.97522 [M − H − 3CH_3_]^−^ 269.98053 [M − H − 3CH_3_ − CO]^−^	D
**59**	Theogallin	6.68	C_14_H_16_O_10_	343.06720 [M − H]^−^	0.38	169.01408 [C_7_H_5_O_5_]^−^ 125.02427 [C_6_H_5_O_3_]^−^	B
**60**	Chlorogenic acid	9.88	C_16_H_18_O_9_	353.08813 [M − H]^−^	0.93	191.05602 [C_7_H_11_O_6_]^−^ 179.0349 [C_9_H_7_O_4_]^−^ 135.04512 [C_8_H_7_O_2_]^−^	B
**61 ^#^**	Vitexin	15.74	C_21_H_20_O_10_	431.09833 [M − H]^−^	−0.1	341.06631 [C_18_H_13_O_7_]^−^ 311.05603 [C_17_H_11_O_6_]^−^ 283.06088 [C_16_H_11_O_5_]^−^	C
**62 ^#^**	9,10-Dihydro-10-(4-hydroxyphenyl)-pyrano[2,3-h]epicatechin-8-one	19.9	C_24_H_20_O_8_	435.10876 [M − H]^−^	0.51	341.06641 [M − H − Phenol]^−^ 217.01392 [C_11_H_5_O_5_]^−^ 189.01918 [C_10_H_5_O_4_]^−^ 177.01915 [C_9_H_5_O_4_]^−^	C
**63**	Epicatechin monogallate	15.92	C_22_H_18_O_10_	441.08286 [M − H]^−^	0.31	289.07166 [C_15_H_13_O_6_]^−^ 169.0141 [C_7_H_5_O_5_]^−^ 125.02431 [C_6_H_5_O_3_]^−^	C
**64**	Astragalin	15.06	C_21_H_20_O_11_	447.09344 [M − H]^−^	0.36	357.06146 [C_18_H_13_O_8_]^−^ 327.05087 [C_17_H_11_O_7_]^−^ 299.05569 [C_16_H_11_O_6_]^−^	C
**65 ^#^**	Kaempferol-3-glucoside	16.47	C_21_H_20_O_11_	447.09354 [M − H]^−^	0.56	285.03983 [M − H − Glc]^−^	C
**66 ^#^**	Luteolin-7-glucoside	17.4	C_21_H_20_O_11_	447.09357 [M − H]^−^	0.63	284.0325 [M − H − Glc]^−^ 255.02986 [C_14_H_7_O_5_]^−^ 227.03485 [C_13_H_7_O_4_]^−^	C
**67 ^#^**	Ursolic acid	35.96	C_30_H_48_O_3_	455.35315 [M − H]^−^	0.18	407.33292 [C_29_H_43_O]^−^	E
**68** *^,**#**^	Oleanic acid	36.51	C_30_H_48_O_3_	455.35333 [M − H]^−^	0.57	407.33292 [C_29_H_43_O]^−^	E
**69 ^#^**	Quercetin-3-alloside	16.21	C_21_H_20_O_12_	463.08856 [M − H]^−^	0.78	300.02731 [M − H − Gal]^−^ 271.02469 [C_14_H_7_O_6_]^−^ 255.02979 [C_14_H_7_O_5_]^−^	C
**70**	Nigranoic acid	32.15	C_30_H_46_O_4_	469.33249 [M − H]^−^	0.33	423.327 [C_29_H_43_O_2_]^−^	B
**71 ^#^**	4-*O*-(6″-*O*-*p*-Coumaroyl-glucopyranosyl)-*p*-coumaric acid	18.64	C_24_H_24_O_10_	471.12994 [M − H]^−^	0.58	307.08206 [C_15_H_15_O_7_]^−^ 163.03993 [C_9_H_7_O_3_]^−^ 119.05008 [C_8_H_7_O]^−^	B
**72**	Corosolic acid	31.81	C_30_H_48_O_4_	471.34802 [M − H]^−^	0.08	453.33932 [C_30_H_45_O_3_]^−^	E
**73 ^#^**	7-Hydroxy-3,8-dimethoxy-5,10-dioxo-5,10-dihydro-chromeno[5,4,3-cde]chromen-2-yl 6-deoxymannopyranoside	18.83	C_22_H_20_O_12_	475.08853 [M − H]^−^	0.7	460.06451 [M − H − CH_3_]^−^ 328.02231 [M – H − Rha]^−^ 312.99887 [M − H − Rha − CH_3_]^−^ 269.98038 [C_13_H_2_O_7_]^−^	D
**74**	Isorhamnetin-7-*O*-glucopyranoside	17.62	C_22_H_22_O_12_	477.10416 [M − H]^−^	0.64	314.04306 [M − H − Glc]^−^ 271.02448 [M − H − Glc − CH_3_ − CO]^−^ 243.02959	C
**75**	Isomyricitrin	14.78	C_21_H_20_O_13_	479.08301 [M − H]^−^	−0.22	316.02216 [M − H − Glc]^−^ 271.02454 [C_14_H_7_O_6_]^−^	C
**76**	1,6-Bis-*O*-galloyl-glucose	11.52	C_20_H_20_O_14_	483.07819 [M − H]^−^	0.33	465.10410 [M − H − H_2_O]^−^ 439.08768 [M − H − CO_2_]^−^	B
**77**	Quillaic acid	26.36	C_30_H_46_O_5_	485.32706 [M − H]^−^	−0.39	407.29538 [M − H − CH_3_ − H_2_O]^−^ 241.10255	E
**78 ^#^**	2-*O*-(*E*)-caffeoyl-1-*O*-*p*-(*E*)-coumaroylglucopyrannose	12.86	C_24_H_24_O_11_	487.12473 [M − H]^−^	0.29	323.07706 [C_15_H_15_O_8_]^−^ 161.02429 [C_9_H_5_O_3_]^−^ 119.05011 [C_8_H_7_O]^−^	B
**79** *^,**#**^	Asiatic acid	26.85	C_30_H_48_O_5_	487.34271 [M − H]^−^	−0.38	469.33224 [M − H − H_2_O]^−^	E
**80**	Oenin	18.16	C_23_H_24_O_12_	491.11981 [M − H]^−^	0.64	313.03531 [M − H − Glc − CH]^−^_3_ 299.01956 [C_15_H_7_O_7_]^−^ 285.04028 [M − H − Glc − CH_3_ − CO]^−^	C
**81**	Medicagenic acid	24.38	C_30_H_46_O_6_	501.32227 [M − H]^−^	0.21	455.31689 [M − H − HCOOH]^−^	E
**82**	2″-*O*-Galloylisovitexin	17.72	C_28_H_24_O_14_	583.10974 [M − H]^−^	0.71	431.09842 [C_21_H_19_O_10_]^−^ 341.06635 [C_18_H_13_O_7_]^−^ 311.056 [C_17_H_11_O_6_]^−^ 283.061 [C_16_H_11_O_5_]^−^	C
**83**	Nicotiflorin	17.02	C_27_H_30_O_15_	593.15167 [M − H]^−^	0.81	285.03989 [C_15_H_9_O_6_]^−^ 255.02983 [C_14_H_7_O_5_]^−^ 227.03491 [C_13_H_7_O_4_]^−^	C
84 *	Rutin	15.9	C_27_H_30_O_16_	609.14630 [M − H]^−^	0.32	300.02737 [C_15_H_8_O_7_]^−^ 271.02469 [C_14_H_7_O_6_]^−^ 255.02977 [C_14_H_7_O_5_]^−^	C
**85**	2′-*O*-Galloylhyperin	15.56	C_28_H_24_O_16_	615.09943 [M − H]^−^	0.44	463.08804 [C_21_H_19_O_12_]^−^ 300.02734 [C_15_H_8_O_7_]^−^ 271.02454 [C_14_H_7_O_6_]^−^ 255.02972 [C_14_H_7_O_5_]^−^	C
**86**	3-*O*-*trans-p*-Coumaroylmaslinic acid	34.83	C_39_H_54_O_6_	617.38501 [M − H]^−^	0.4	145.02936 [C_9_H_5_O_2_]^−^	E
**87**	Delphinidin-3-caffeoylglucoside	18.25	C_30_H_26_O_15_	625.12048 [M − H]^−^	0.94	463.08832 [C_21_H_19_O_12_]^−^ 300.02731 [C_15_H_8_O_7_]^−^ 271.02472 [C_14_H_7_O_6_]^−^ 255.02985 [C_14_H_7_O_5_]^−^	C
**88**	3-*O*-*trans-p*-Coumaroyltormentic acid	31.21	C_39_H_54_O_7_	633.37982 [M − H]^−^	0.23	163.04001 [C_9_H_7_O_3_]^−^ 145.02939 [C_9_H_5_O_2_]^−^	E
**89**	1,3,6-Tri-*O*-galloylglucose	14.4	C_27_H_24_O_18_	635.08948 [M − H]^−^	0.77	465.06714 [C_20_H_17_O_13_]^−^ 211.02463 [C_9_H_7_O_6_]^−^ 169.01404 [C_7_H_5_O_5_]^−^ 125.02427 [C_6_H_5_O_3_]^−^	B
**90**	(3,5,9)-3-Hydroxy-27-{[(2*E*)-3-(4-hydroxy-3-methoxyphenyl)-2-propenoyl]oxy}olean-12-en-28-oic acid	35.86	C_40_H_56_O_7_	647.39587 [M − H]^−^	0.84	632.37152 [C_37_H_52_O_7_]^−^ 453.33835 [C_30_H_45_O_3_]^−^ 175.03993 [C_10_H_7_O_3_]^−^ 133.02946 [C_8_H_5_O_2_]^−^	E
**91**	3-*O*-*trans*-Feruloyleuscaphic acid	32.02	C_40_H_56_O_8_	663.39069 [M − H]^−^	0.67	648.36658 [C_39_H_52_O_8_]^−^ 175.03989 [C_10_H_7_O_3_]^−^ 160.01643 [C_9_H_4_O_3_]^−^ 132.02156 [C_8_H_4_O_2_]^−^	E
**92 ^#^**	4′-Hydroxyacetophenone	12.32	C8H8O2	137.05969 [M + H]^+^	−0.01	122.03635 [M + H − CH_3_]^+^ 109.06508 [M + H − CO]^+^	F
**93**	Dihydroxyacetophenone	13.7	C_8_H_8_O_3_	153.05461 [M + H]^+^	−0.06	125.05981 [M + H − CO]^+^ 111.04433 [C_6_H_7_O_2_]^+^	F
**94**	*N*-Lauryldiethanolamine	37.04	C_16_H_35_O_2_N	256.26334 [M + H]^+^	−0.6	144.1382 [C_8_H_18_ON]^+^ 116.1072 [C_6_H_14_ON]^+^	F
**95**	Licanic acid	26.99	C_18_H_28_O_3_	293.21085 [M + H]^+^	−0.94	275.20044 [M + H − H_2_O]^+^ 257.19003 [M + H − 2H_2_O]^+^	A
**96**	Kamlolenic acid	33.51	C_18_H_30_O_3_	295.22678 [M + H]^+^	0.06	277.21609 [M + H − H_2_O]^+^ 259.20532 [M + H − 2H_2_O]^+^ 231.21051 [M + H − 3H_2_O]^+^	A
**97**	Diosmetin	21.99	C_16_H_12_O_6_	301.0705 [M + H]^+^	−0.56	286.04681 [M + H − CO_3_]^+^ 258.05197 [M + H − CH_3_ − CO]^+^	C
**98 ^#^**	Sitosterol	39.21	C_29_H_50_O	397.38269 [M + H − H_2_O]^+^	−0.47	243.21089 [C_18_H_27_]^+^ 175.14799 [C_13_H_19_]^+^ 147.11684 [C_11_H_15_]^+^	G
**99 ^#^**	Stigmasterol	38.08	C_29_H_48_O	395.36725 [M + H − H_2_O]^+^	0.05	241.1945 [C_18_H_25_]^+^ 199.14772 [C_15_H_19_]^+^ 173.13248 [C_13_H_17_]^+^	G
**100**	Apigenin-7-*O*-glucoside	17.83	C_21_H_20_O_10_	433.1127 [M + H]^+^	−0.51	271.05975 [C_15_H_11_O_5_]^+^ 153.01814 [C_7_H_5_O_4_]^+^	C
**101 ^#^**	Quercetin-3-arabinoside	16.62	C_20_H_18_O_11_	435.09235 [M + H]^+^	0.36	303.04959 [C_15_H_11_O_7_]^+^ 153.01819 [C_7_H_5_O_4_]^+^	C
**102**	Luteolin-7-galactoside	12.52	C_21_H_20_O_11_	449.10745 [M + H]^+^	−0.86	287.05453 [M − H − Glc]^+^ 269.044 [M + H − Glc]^+^	C
**103**	Pelargonidin-3-*O*-(6-caffeoyl-glucoside)	19.91	C_30_H_26_O_13_	595.14459 [M + H]^+^	−0.04	433.11255 [M − H − C_6_H_6_ − 3CO]^+^ 313.07028 [C_17_H_13_O_6_]^+^ 163.03888 [C_9_H_7_O_3_]^+^	C
**104**	Tiliroside	20.42	C_30_H_26_O_13_	595.14435 [M + H]^+^	−0.45	287.0546 [C_15_H_11_O_6_]^+^ 147.04393 [C_9_H_7_O_2_]^+^ 119.04929 [C_8_H_7_O]^+^	C
**105**	Kaempferol-3-(6″-galloylgalactoside)	16.8	C_28_H_24_O_15_	601.11914 [M + H]^+^	0.57	287.05457 [C_15_H_11_O_6_]^+^ 153.01813 [C_7_H_5_O_4_]^+^ 137.0233 [C_7_H_5_O_3_]^+^	C
**106**	Quercetin-3-*O*-(6″-*O*-p-coumaroyl)-glucopyranoside	19.41	C_30_H_26_O_14_	611.13922 [M + H]^+^	−0.51	147.04388 [C_9_H_7_O_2_]^+^ 303.04953 [C_15_H_11_O_7_]^+^	C
**107**	2′-*O*-Galloylhyperin	15.51	C_28_H_24_O_16_	617.11322 [M + H]^+^	−0.89	153.01813 [C_7_H_5_O_4_]^+^ 303.0495 [C_15_H_11_O_7_]^+^	C
**108**	Quercetin-3-(6″-caffeoylgalactoside)	18.29	C_30_H_26_O_15_	627.13409 [M + H]^+^	−0.56	163.03873 [C_9_H_7_O_3_]^+^ 303.04941 [C_15_H_11_O_7_]^+^	C
**109 ^#^**	Casuarinin	12.61	C_41_H_28_O_26_	937.09332 [M + H]^+^	−0.89	153.01817 [C_7_H_5_O_4_]^+^ 171.04417 [C_11_H_7_O_2_]^+^ 277.03397 [C_13_H_9_O_7_]^+^ 345.02377 [C_16_H_9_O_9_]^+^	D

* These compound were unambiguously identified by the use of authentic reference compounds. **^#^** These compound were isolated from *Melastoma dodecandrum* Lour. according to the literature [5,6,7,8,9,10,11,12,13]. Glc, glucopyranosyl, Rha, rhamnopyranosyl. A, fatty acid; B, organic acid; C, flavonoid; D, tannin; E, pentacyclic triterpene; F, others; G, steroid.

**Table 2:** RDA fragmentation pathways of compound **29**, **30**, **46**, and **63**.

Compound No.	Mass Spectra of MS/MS	RDA Fragmentation Pathway
**29**	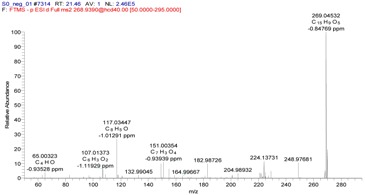	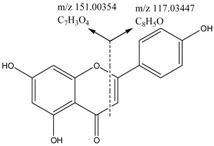
**30**	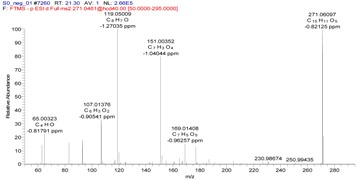	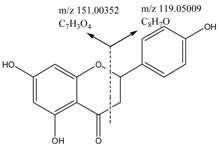
**46**	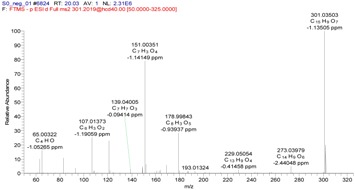	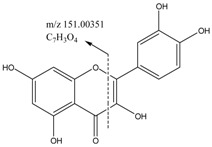
**63**	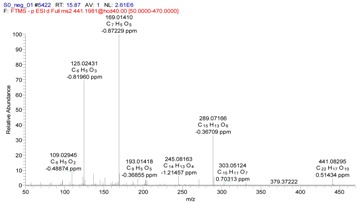	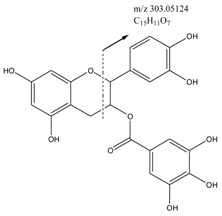

## References

[1] Zhang Z., Wang X., Wang J., Jia Z., Liu Y., Xie X., Wang C., Jia W. (2016). Metabonomics Approach to Assessing the Metabolism Variation and Endoexogenous Metabolic Interaction of Ginsenosides in Cold Stress Rats. J. Proteome Res..

[2] Zhang Z.H., Vaziri N.D., Wei F., Cheng X.L., Bai X., Zhao Y.Y. (2016). An integrated lipidomics and metabolomics reveal nephroprotective effect and biochemical mechanism of *Rheum officinale* in chronic renal failure. Sci. Rep..

[3] Yang Y., Zhang Z., Li S., Ye X., Li X., He K. (2014). Synergy effects of herb extracts: Pharmacokinetics and pharmacodynamic basis. Fitoterapia.

[4] Yu Z.C., Lin X.X., Su J.Q., Lin Q.J., Chen Z.D. (2011). Advance in *Melastoma dodecandrum* Lour. Researches. Med. Plant.

[5] Zhang R.Z. (2013). Studies on the Chemical Constituents of *Melastoma dodecandrum* L. and *Achillea alpine*. Master’s Thesis.

[6] Cheng M. (2015). Studies on the Chemical Constituents of *Melastoma dodecandrum* L.. Master’s Thesis.

[7] Lin S., Li Y.C., Guo Y.Y., Guo S.M., Que H.Q., Qi Y.P. (2009). Chemical constituents of *Melastoma dodecandrum* L. (II). Chin. Tradit. Herb. Drugs.

[8] Tang M., Liao B.Z., Lin S., Deng S.S. (2008). Chemical constituents of *Melastoma dodecandrum* L.. Chin. Tradit. Herb. Drugs.

[9] Zeng R.X., Zhang Q.H., Guan Y.M., Chen L.H., Wen S.J., Lu X.P. (2015). Analysis of monosaccharide composition in polysaccharides extract from *Melastoma dodecandrum* L. by precolumn derivatization HPLC. Chin. J. Exp. Tradit. Med. Formulae.

[10] Cao D., Ma Z.Q., Jiang Y., Zhao C.J., Lin R.C. (2016). Chemical Composition of *Melastoma dodecandrum* Lour.. Inf. Tradit. Chin. Med..

[11] Yang D., Ma Q.Y., Liu Y.Q., Ding Z.T., Zhou J., Zhao Y.X. (2010). Chemical Constituents from *Melastoma dodecandrum* Lour.. Nat. Prod. Res. Dev..

[12] Zhang C., Fang Y.X. (2003). Studies of the Chemical Constituents of Chinese Herb *Melastoma dodecandrum* Lour.. China J. Chin. Mater. Media.

[13] Zhang C. (2003). Studies on Chemical Constituents and Pharmacological Activities of Chinese Herbal Melastoma dodecandrum Lour.. Master’s Thesis.

[14] Kouloura E., Skaltsounis A.L., Michel S., Halabalaki M. (2015). Ion tree-based structure elucidation of acetophenone dimers (AtA) from *Acronychia pedunculata* and their identification in extracts by liquid chromatography electrospray ionization LTQ-Orbitrap mass spectrometry. J. Mass Spectrom..

[15] Zhang J.Y., Wang F., Zhang H., Lu J.Q., Qiao Y.J. (2014). Rapid identification of polymethoxylated flavonoids in traditional Chinese medicines with a practical strategy of stepwise mass defect filtering coupled to diagnostic product ions analysis based on a hybrid LTQ-Orbitrap mass spectrometer. Phytochem. Anal..

[16] Ru L., Wei S., Xue Q., Jia L., Hong L., Min Y. (2015). Chemical profiling of *Scutellaria barbata* by ultra high performance liquid chromatography coupled with hybrid quadrupole orbitrap mass spectrometry. J. Chin. Pharm. Sci..

[17] Dong H.J., Chen X.H., Zeng R. (2016). Rapid analysis on chemical constituents in roots of *Rheum pumilum* by UPLC coupled with hybrid quadrupole-orbit trap MS. Chin. Tradit. Herb. Drugs.

[18] Wang S.S., Xu H.Y., Ma Y.N., Wang X.G., Shi Y., Huang B., Tang S.H., Zhang Y., Li D.F., Liang R.X. (2015). Characterization and rapid identification of chemical constituents of NaoXinTong capsules by UHPLC-linear ion trap/Orbitrap mass spectrometry. J. Pharm. Biomed. Anal..

[19] Zhao Y.Y., Cheng X.L., Zhang Y., Chao X., Zhao Y., Lin R.C., Sun W.J. (2010). A fast and sensitive HPLC-MS/MS analysis and preliminary pharmacokinetic characterization of ergone in rats. J. Chromatogr. B Anal. Technol. Biomed. Life Sci..

[20] Zhao Y.Y., Qin X.Y., Cheng X.L., Liu X.Y., Lin R.C., Zhang Y., Li X.Y., Sun X.L., Sun W.J. (2010). Rapid resolution liquid chromatography-mass spectrometry and high-performance liquid chromatography-fluorescence detection for metabolism and pharmacokinetic studies of ergosta-4,6,8(14),22-tetraen-3-one. Anal. Chim. Acta.

[21] Guo L.X., Li R., Liu K., Yang J., Li H.J., Li S.L., Liu J.Q., Liu L.F., Xin G.Z. (2015). Structural characterization and discrimination of Chinese medicinal materials with multiple botanical origins based on metabolite profiling and chemometrics analysis: Clematidis Radix et Rhizoma as a case study. J. Chromatogr. A.

[22] Mao Q., Bai M., Xu J.D., Kong M., Zhu L.Y., Zhu H., Wang Q., Li S.L. (2014). Discrimination of leaves of *Panax ginseng* and *P. quinquefolius* by ultrahigh performance liquid chromatography quadrupole/time-of-flight mass spectrometry based metabolomics approach. J. Pharm. Biomed. Anal..

[23] Duan L., Guo L., Liu K., Liu E.H., Li P. (2014). Characterization and classification of seven citrus herbs by liquid chromatography-quadrupole time-of-flight mass spectrometry and genetic algorithm optimized support vector machines. J. Chromatogr. A.

